# Factor mobility, transportation network and green economic growth of the urban agglomeration

**DOI:** 10.1038/s41598-022-24624-5

**Published:** 2022-11-22

**Authors:** Yuqi Yang, Xiangyi Lu, Jun Chen, Na Li

**Affiliations:** 1 School of Marxism, China University of Geosciences (Wuhan), Wuhan, 430074 Hubei China; 2 School of Economics and Management, China University of Geosciences (Wuhan), Wuhan, 430074 Hubei China; 3 Resource and Environment Center, China University of Geosciences (Wuhan), Wuhan, 430074 Hubei China

**Keywords:** Environmental sciences, Environmental social sciences

## Abstract

Understanding the externalities of transportation networks in the process of the agglomeration and diffusion of production factors has theoretical and practical significance for the coordinated development of China's economic growth in urban agglomerations. Therefore, the social network analysis method is introduced in this paper with the case of the Pan Pearl River Delta Urban Agglomeration to analyze the characteristics of the traffic connection network of the production factor flow within this urban agglomeration, and subsequently, an econometric panel model is adopted to quantitatively analyze the effect of the connection network on the economic growth of the urban agglomeration. The results show that (1) the traffic connection of the Pan Pearl River Delta Urban Agglomeration has network characteristics typical of a “small world”. Although the connections between cities are gradually strengthening, the regional differences are obvious, showing a core–edge pattern of eastern agglomeration and western sparseness. (2) Among the network nodes, Guangzhou, Shenzhen and other cities have obvious agglomeration and diffusion effects, stabilizing economic growth while driving the development of surrounding cities. The "polarization effect" in Chongqing and Chengdu has significantly increased, and the accumulation of factors mainly meets their own economic development but has not yet spread. (3) The Pan Pearl River Delta Urban Agglomeration's transportation network influences the region’s economic growth through the structural effect, as it strengthens the economic ties between cities, and through the action of resource factors, as the network represents the aggregation and diffusion path of factor flow. (4) Due to the different traffic connections and industrial structures across the Pan Pearl River Delta Urban Agglomeration, the factor flow of each suburban agglomeration has a differentiated impact on the regional economic growth under the traffic connection network. Therefore, to realize the coordinated economic development of the Pan Pearl River Delta Urban Agglomeration, it is necessary to "adjust measures to local conditions" and formulate accurate and precise policies.

## Introduction

Over the past 40 years of reform and opening up, China has gradually formed urban agglomerations such as the Yangtze River Delta, Beijing–Tianjin–Hebei and Pearl River Delta, which have become the important growth poles of China's economy and reshaped the spatial pattern of economic development^1^. These urban agglomerations have become the hinterland of China's highly concentrated economic activities. The formation of urban agglomerations depends on a high degree of interconnection between urban transportation networks, but the impact of developed transportation networks on the economic growth of urban agglomerations has two sides^2^: the radiation effect and the polarization effect^3^. The “radiation effect” means that traffic reduces the transaction costs of production factors, accelerates the flow of labor and resources within and between urban agglomerations, and causes developed areas to have a driving effect on the economic development of surrounding areas^4^. The polarization effect means that the transportation network intensifies the polarized allocation of the internal elements of urban agglomeration, leading to the agglomeration of production resources in the central city in the network, resulting in the unbalanced spatial allocation of elements and further widening the economic gap between the central city and the surrounding cities^5^. Strengthening the "radiation effect" of traffic networks on the economic growth of urban agglomerations and reducing the "polarization effect" are the key issues in the process of realizing the integrated development of urban agglomerations.

An urban agglomeration is a group of cities with compact spatial organization, close economic ties and high degree of urbanization and integration, which is based on a developed transportation and communication network^6^. The greatest advantage of the development of an urban cluster economy is the formation of scale economies through the cooperation of industries among cities, resulting in the effect of "1 + 1 > 2". However, at present, many urban agglomerations have a "center-periphery" spatial structure, which leads to economic inequality^7,8^. A large number of studies have analyzed the causes of inequality in urban agglomerations from the perspectives of traffic network structure^9^, urban competitiveness^10^, regional industrial competition^11,12^, and social relations^13^. Since the self-organization process of urban agglomerations depends on large-scale transportation infrastructure investment, the influence of transportation networks on the economic growth of urban agglomerations has been widely discussed.

Inequality in the network comes from the polarized allocation of factors by network structures^14^ and from the unequal gravity between nodes and factors^13^. This is mainly related to two research fields: first, exploring the optimization direction of network structure from the perspective of improving the efficiency or fairness of network configuration; and second, finding a reference from research on the relationship between factor mobility and economic growth. The former research mostly focuses on the congestion and efficiency loss caused by the imperfect network structure, thus proposing the construction scheme of future networks^15,16^. The latter regards the transportation network as an exogenous environment and then discusses how to make decisions to maximize the benefits in the existing network^17–19^. In fact, these two kinds of studies together constitute a system of solutions to alleviate the economic inequality in the network. However, the investment in transportation infrastructure is irreversible, and the existing network structure is difficult to change. It takes a long time and large investment to improve resource allocation from the perspective of network structure optimization. Therefore, to achieve equitable economic growth of urban agglomerations, the construction of regional coordination mechanisms in the network should be considered. This requires a deep understanding of the internal relationship among the traffic network, factor flow and economic growth of urban agglomerations.

Based on the above discussion, this paper attempts to answer the following three questions: First, how can the urban correlation strength on the traffic network be described from the perspective of factor flow? Second, how does the transportation network affect the economic development of urban agglomerations? Third, what measures should be taken by local governments in an urban agglomeration to promote the coordinated development within the agglomeration?

The specific research contents and objectives are outlined as follows: Taking China's Pearl River Delta urban agglomeration as the research object and using the measurement method of urban flow intensity, the study depicts the traffic connection network, which includes factor flow, economic connection and geographical space abstraction. Then, the social network analysis method is used to analyze the overall and local characteristics of the transportation network from the two dimensions of network structure and network nodes to reveal the influence path of urban agglomeration traffic-related networks on resource allocation and economic growth. Finally, regression analysis is used to identify the key factors of the economic growth of urban agglomerations under network constraints. Based on the results, we put forward some suggestions for the coordinated development of urban agglomerations.

The contributions are as follows: (1) As a case study, from the perspective of the traffic network and factor flow, we put forward suggestions for the coordinated development of the regional economy in the Pearl River Delta Urban Agglomeration and similar urban agglomerations. (2) In a wider sense, the construction of a generalized traffic connection network including factor flow, economic connection and geographical distance provides a new research method to reveal the internal power driving the economic growth of urban agglomerations, which provides a reference for related research.

## Model and data

### Association network model of factor flow

Gravity models often use GDP and population as the "quality" to describe the relationship between different economies^20^. However, the agglomeration and diffusion of resources flowing between urban agglomerations are complex, frequent and multidirectional, covering the transfer and exchange of many elements, such as people, logistics, and information flow, among cities. Obviously, GDP and population reflect the development level of the urban economy, but they lack a description of the path and intensity of multidimensional factor transfer and cannot describe the state of agglomeration and radiation in the process of factor flow. Therefore, this paper chooses the intensity of urban flow as the "quality" of the economy to express the overall development level of cities in urban agglomerations, which not only reveals the constant exchange of material and energy between cities but also more clearly depicts the characteristic that the transportation network supports the communication between cities^21,22^. Considering that the transportation network of urban agglomerations is mainly composed of roads and rails, so the volume of freight and passengers objectively reflects the flow forms of production factors among urban agglomerations, the traffic network correlation value is finally obtained:

1 $$ F_{ij} = K_{ij} \sqrt {E_{i} N_{i} \times E_{j} N_{j} } /D_{ij}^{2} $$

2 $$ K_{ij} = \left[ {W_{1} \left( {\frac{{Q_{i1} }}{{Q_{i1} + Q_{j1} }}{ + }\frac{{C_{i1} }}{{C_{i1} + C_{j1} }}} \right) + W_{2} \left( {\frac{{Q_{i2} }}{{Q_{i2} + Q_{j2} }} + \frac{{C_{i2} }}{{C_{i2} + C_{j2} }}} \right)} \right]/2 $$

where $F_{ij}$ is the intensity of the traffic correlation network of city $i$ and city $j$, $N_{i}$ and $N_{j}$ are the urban functional benefits of city $i$ and city $j$, respectively, and $E_{i}$ and $E_{j}$ are the outward functional quantities of city $i$ and city $j$, respectively. See^22^ for the specific algorithm of the above two variables. $D_{ij}$ represents the shortest spatial distance between two cities. $k_{ij}$ represents the traffic connection coefficient between city $i$ and city $j$, where $Q_{i1}$ and $Q_{j1}$, respectively, represent the freight volume of roads in $i$ and $j$,$Q_{i1}$ and $Q_{j1}$, respectively, represent the freight volume of rails in $i$ and $j$; $c_{i2}$ and $c_{j2}$, respectively, represent the passenger volume of roads in $i$ and $j$; $Q_{i2} ,Q_{j2}$, respectively, represent the passenger volume of rails in $i$ and $j$; and $W_{1}$ and $W_{2}$, respectively, represent the weights of roads and rails.

On the basis of constructing a traffic correlation matrix by a gravity model, social network analysis (SNA) can be used to analyze the traffic correlation network structure. Common indicators of overall network structure include network density, clustering coefficient and characteristic path length. Among them, network density measures the closeness of the connection between cities. The greater the density is, the more ways to exchange information between cities and the more efficient the communication. The clustering coefficient reflects the node degree and node aggregation in the network, and aggregation means that the probability of community formation increases. The average shortest path indicates the rate and average cost of element flow in the network. The smaller the average path is, the less time it takes for element flow and the lower the transportation cost^23^. These three indicators can reflect the scale and efficiency of the factor flow within the urban agglomeration when applied to the analysis of the urban agglomeration traffic network structure, thus revealing the supporting role of the network for the coordinated economic growth among cities.

This paper analyzes the network structure characteristics of each node through centrality and reveal the central position of node cities in the network structure^24^. Centrality includes degree centrality (in-degree and out-degree), betweenness centrality and closeness centrality. The higher the degree centrality is, the more connections the city has with other cities in the traffic spatial correlation network, and the more central the city is in the network. Betweenness degree measures the ability of nodes as mediators, which indicates the extent to which the city can control the traffic exchanges between other cities. The closeness centrality describes the degree to which a city in the network approaches the center in the transportation network and reflects the center-edge structure. The closer a city is to the central node, the stronger its role in resource allocation in the network.

### The model of network effects on economic growth

This paper focuses on verifying the structural effect of the traffic connection network structure in urban agglomerations, so it tries to analyze the mechanism by which the structural effect of the traffic connection network structure influences the economic growth of urban agglomerations from the two dimensions of the overall structural effect and individual structural effect of the traffic connection network.

#### Network structure effect model

The coordinated development of urban agglomerations is the ultimate result pursued in the economic growth of urban agglomerations, so this paper chooses the indicator of the coordinated development of urban agglomerations^25^ as the explained variable (Formula 3) to express the close economic connection and dependence among cities within urban agglomerations. The network density, aggregation coefficient and characteristic path length are taken as the explained variables, univariate OLS regression is performed, and the explained variables and the explained variables are logarithmically processed. The formula is as follows:

3 $$ \ln Z_{1} = \alpha_{0} + \beta \ln (x) $$

4 $$ Z_{1} = \frac{n}{{\sum\limits_{i = 1}^{n} {\sum\limits_{j = 1}^{n} {W_{ij} } } }} \times \frac{{\sum\limits_{i = 1}^{n} {\sum\limits_{j = 1}^{n} {W_{ij} (x_{i} - \overline{x} )(x_{j} } } - \overline{x} )}}{{\sum\limits_{i = 1}^{n} {(x_{i} - \overline{x} )^{2} } }} $$

where $Z_{1}$ is the intercity connectivity, and $x_{i}$ represents the per capita GDP of city $i$ in that year; $n$ is the number of cities within the urban agglomeration; $\overline{x}$ represents the average GDP per capita of $n$ cities in the current year; and $W_{ij}$ represents the element in the spatial adjacent weight matrix between two cities. This paper does not limit the adjacent edge limitation of traditional spatial econometrics. In dealing with the weight coefficient, relying on the simulation and characteristics of the attenuation function of traffic flow in China, we choose the best Pareto model of the distance attenuation function^26^, $W_{ij} = ae^{{b\ln d_{ij} }}$, and both $a$ and $b$ are coefficients.

#### Network node effect model

The factors that influence regional economic growth are very complicated, and economic growth may be achieved with the spatial synergy of multidimensional factors. Due to the flow of production factors, the aggregation effect and the demonstration effect of economic growth, the economic growth of different regions also has heterogeneity. Therefore, in order to explore the aggregation and diffusion effects of factor flow on the regional economy and regional differences under the traffic connection network structure, this paper decomposes the overall network structure effect into nodes, constructs a benchmark panel model of factor flow on economic growth under the nontraffic connection network structure, and on this basis, adds the structural factors of individual structure network traffic connection network to analyze how the traffic connection network affects the factor flow and promotes the economic growth of the urban agglomeration. It then determines the economic cross oaf different regions by deconstructing the Pan Pearl River Delta Urban Agglomeration.

Assuming that each city follows the following logarithmic linear production function, a panel data model is constructed without considering the traffic correlation network. Because there are great individual differences and time series differences among the whole urban agglomeration and suburban agglomerations, to control the heterogeneity between individuals and time and simplify the processing, this paper adopts a panel model of with individual and time fixed effects. The benchmark model is shown in Formula 5, and the structural variables of individual networks are introduced to investigate the structural effects of related networks on the economic growth of urban agglomerations. Therefore, key indicators of nodes are added in Formula 5, and the established model is shown in Formula 6.

5 $$ \ln Y_{p} = \alpha_{0} + \beta_{1} {\text{lnSIR}} + \beta_{2}\, {\text{lnTIR}} + \beta_{3} \ln FDI + \beta_{4} \ln UR{ + }\eta_{i} { + }\nu_{t} { + }\varepsilon_{1} $$

6 $$ \begin{gathered} \ln Y_{P} = \alpha_{0} { + }\beta_{1} \ln SIR{ + }\beta_{2}^{^{\prime}} \ln TIR{ + }\beta_{3}^{^{\prime}} \ln FDI{ + }\beta_{4}^{^{\prime}} \ln UR{ + }\beta_{5}^{^{\prime}} \ln OD + \beta_{6}^{^{\prime}} \ln ID + \beta_{7}^{^{\prime}} \ln BT + \beta_{8}^{^{\prime}} \ln CL{ + } \hfill \\ \eta_{i} { + }\nu_{t} { + }\varepsilon_{2} \hfill \\ \end{gathered} $$

Industrial emission data represent green elements,

where $Y_{p}$ is the per capita GDP, and the industrial structure (SIR- proportion of secondary industry output value to GDP; TIR- proportion of tertiary industry output value to GDP), foreign direct investment (FDI) and urbanization rate (UR- proportion of urban population to total population) are the explanatory variables, which are specifically explained as follows. ① Industrial structure: the transformation of economic growth mode is formed in the process of industrial structure optimization caused by the production and flow of advanced production factors. For a given capital, labor and technology, different industrial structures lead to different production modes and economic directions^27^. ② Foreign direct investment: As the input of capital elements, foreign direct investment reflects the communication between cities and the outside world, and its spillover effect can promote exports, strengthen the economic, technological and management strength of enterprises, improve the quality of production factors, and then accelerate economic growth^28^. ③ Urbanization rate: Urbanization means that the population elements caused by industrialization and modernization are concentrated in cities, and other elements are likewise concentrated and redistributed in space, thus becoming an important driving force for economic growth^29^. This paper takes the proportion of the nonagricultural population among permanent residents at the end of the year as the index of the urbanization rate. The network index ID is the in-degree, OD is the out-degree, BT is betweenness centrality, and CL is closeness centrality. $\eta_{i}$ represents the individual fixed effect of different cities in the panel data, $v_{t}$ represents the time fixed effect, and $\varepsilon_{2}$ represents the random error. The parameters $\beta_{1}$, $\beta_{2}$, $\beta_{3}$, and $\beta_{4}$ reflect the influence of factor flow on economic growth, and $\beta_{5}$, $\beta_{6}$, $\beta_{7}$, and $\beta_{8}$ reflect the influence of network structure on economic growth. When a parameter is positive, it can promote economic growth. If its value is significantly negative, it has a negative inhibitory effect on economic growth.

## Research object and data source

Obviously, the above models and methods are more applicable in urban agglomerations with denser networks. As a typical mega-city group in China, the Pan Pearl River Delta Urban Agglomeration provides an excellent object for us to analyze the relationship between traffic networks and economic growth. The Pan Pearl River Delta Urban Agglomeration is composed of Guangdong, Fujian, Sichuan, Hunan, Yunnan, Hainan, Guizhou, Guangxi Provinces and the Hong Kong Special Administrative Region, spanning the southern, central and western parts of China. The urban agglomeration covers six suburban agglomerations and has gradually formed an engine of future economic development in the delta region^30^. Compared with the Beijing-Tianjin-Hebei and Yangtze River Delta Urban Agglomerations, the Pearl River Delta Urban Agglomeration consists of several suburban agglomerations, the economic level of the suburban agglomerations is quite different, and the urban agglomerations are at different stages of development. For example, the Pan Pearl River Delta Urban Agglomeration has formed a multicore network model, the Chengdu-Chongqing Urban Agglomeration has formed a typical dual-core model, and there is a single-core model with Kunming as the center in central Yunnan^11,12^. In the Pearl River Delta region, there is not only an imbalance of economic development within the suburban agglomerations caused by the agglomeration and diffusion of elements within the suburban agglomerations but also an imbalance of economic development among suburban agglomerations caused by the agglomeration of elements generated by competition among urban agglomerations. Therefore, to study the relationship between the economic growth of urban agglomerations and coordinated development among regions, this paper chooses the Pan Pearl River Delta Urban Agglomeration as the research object.

The sample data in this paper span the period from 2006 to 2015. When calculating the intensity of urban flow, the original data mainly come from the local statistical yearbooks of 9 provinces and 28 cities, which specifically include the data of employees, total personnel and GDP of 16 sectors, including manufacturing, construction, wholesale and retail, transportation, warehousing and postal services, accommodation, catering, finance, education, real estate, etc., which have strong outward functions in the secondary industry. The data of highway passenger traffic and freight volume, railway passenger traffic and freight volume, and resident population at the end of the year involved in calculating the relationship value of the traffic correlation network come from the statistical yearbooks of various provinces and cities and the bulletin of national economic and social development. Among them, the interpolation method is adopted to fill gaps in the freight volume data in some cities. Because the traffic in this paper involves roads and railways, considering that the railway distance data between two places are difficult to obtain, this paper uses the shortest road driving distance between two cities in Baidu map as the spatial distance. Descriptive statistics of the variables are shown in Table 1.

**Table 1:** Descriptive statistics of the variables.

Variable	Unit	Observations	Mean	SD	Min	Max
**Number of employees**	10 thousand					
Mining		280	9.2374	6.5965	0.8010	24.5002
Manufacturing		280	130.7909	149.6594	6.9030	1020.2101
Building		280	48.8414	36.2796	4.5002	177.8021
Traffic, warehousing and postal services		280	20.0222	14.1516	3.6102	83.3041
Communication, computer service and software		280	4.7667	4.1145	0.6101	19.7005
Wholesale and retail trade		280	17.4980	13.5715	2.8002	95.5030
Finance		280	13.2849	9.9551	1.7020	47.6003
Realty		280	7.3323	8.2026	1.1002	52.5103
R&D		280	6.7198	3.9811	1.4021	18.6004
Education		280	56.2748	26.0964	8.4002	118.6003
GDP	100 Million Yuan	280	15,610.58	14,700.003	1052.85	72,812.55
Population	10 thousand people	280	5203.7531	2583.7950	836.0210	10,849.003
**Volume of freight**	10 thousand tons					
Road		280	86,190.923	57,238.557	7981.0031	261,273.02
Rail		280	5752.3726	2374.7025	542.0032	9711.0211
**Volume of passengers**	10 thousand people					
Road		280	106,764.94	105,085.92	10,363.002	556,510.01
Rail		280	5706.3787	4323.0032	23.0024	23,149.110
Urban flow intensity		280	749.9186	810.0310	57.1435	4612.863

## The characteristics of the urban flow network in the Pan Pearl River Delta urban agglomeration

### Temporal and spatial characteristics of the urban flow network

**Figure 1:**
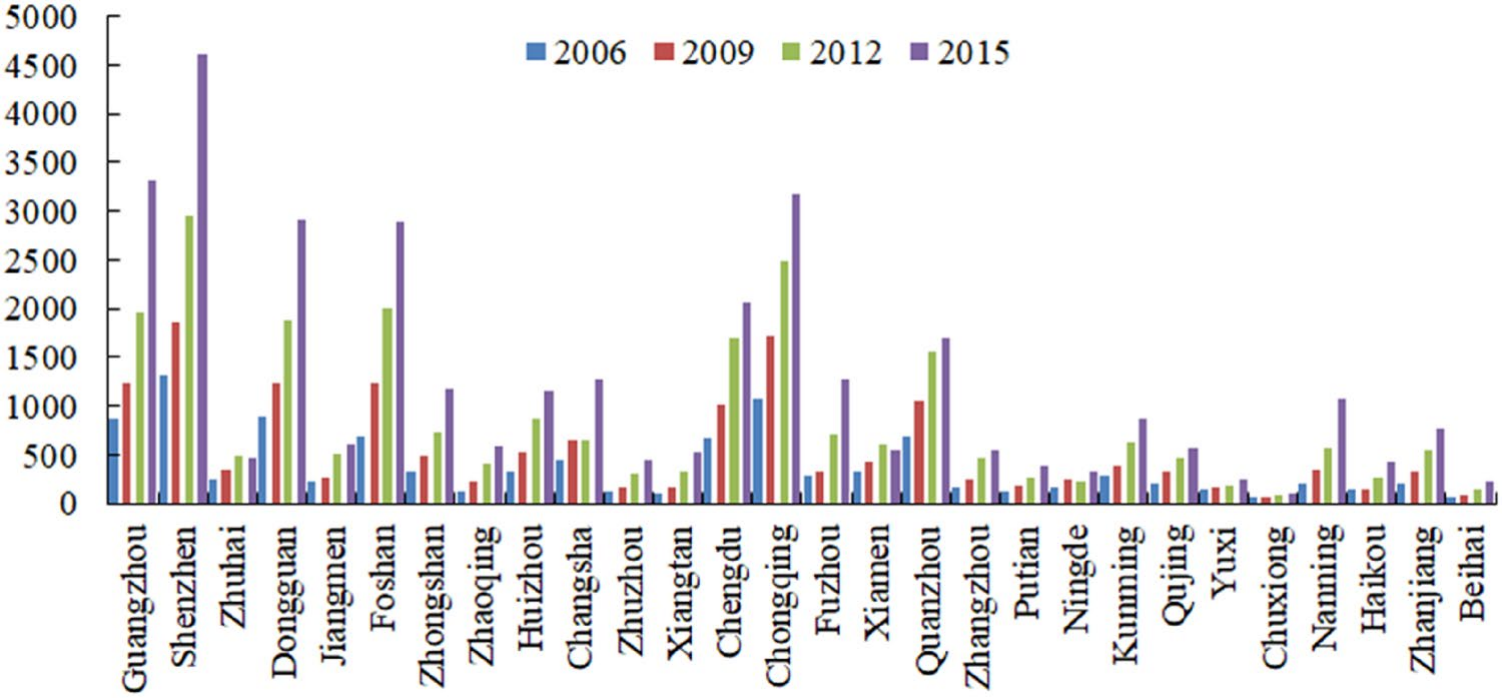
The urban flow density of the Pearl River Delta (2006, 2009, 2012, 2015).

Based on the intensity of urban flows in 2006, 2010, 2012 and 2015 (Fig. 1), this paper discusses the degree of external relations among cities and depicts the characteristics of the flow of resource elements within the Pan Pearl River Delta Urban Agglomeration, in which industrial agglomeration promotes the spatial aggregation and diffusion of elements.

From the overall dynamic change in the urban agglomeration, the intensity of urban flow in cities of the Pearl River Delta urban agglomeration is on the rise, and urban communication is gradually enhanced, but cities present different degrees of communication with the outside world. From the inside of the urban agglomeration, the intensity of urban flow in the Pearl River Delta Urban Agglomeration is much higher than that in other urban agglomerations, which indicates that the cities in the Pearl River Delta Urban Agglomeration are more closely connected with the surrounding external regions and the factors of production can flow frequently among the cities in the urban agglomeration. As a major economic province in China, Guangdong has a large economic output and a high level of development, and the intensity of urban flow in Guangzhou and Shenzhen is the highest, reaching 3317 and 4612, respectively, in 2015, and making these the core leaders of the region with an obvious diffusion effect. There are cities with a high intensity of urban flow in the Sichuan-Chongqing Urban Agglomeration and Beibu Gulf Urban Agglomeration, such as Chongqing, Chengdu, Fuzhou, etc. While the economic strength is obviously enhanced, the status of core cities has been consolidated, which is mainly manifested by the effect of aggregation factor flow, but the radiation capacity is limited. The intensity of urban flow in the central Yunnan urban agglomeration and the urban agglomeration on the west coast of the Taiwan Strait is generally low, which is closely related to the development of cities within the urban agglomeration. Although there is an obvious growth trend in the internal relations of urban agglomerations, the circulation and transmission efficiency of factors is still not high. Generally, there is a large gap between the urban flow intensity of the marginal cities and the core cities, which leads to the limited ability of less-developed cities to take advantage of the economic radiation of the core cities and makes it difficult for the marginal areas of the whole urban agglomeration to enjoy the development achievements of the central cities.

### The evolution of the structure of the urban flow network

After constructing the traffic correlation network matrix by combining the urban flow intensity and gravity model, this paper uses Ucinet 6.0 to obtain the evolution diagram of the traffic correlation network structure of the Pearl River Delta Urban Agglomeration and calculates the overall network characteristic value and individual network centrality.

**Figure 2:**
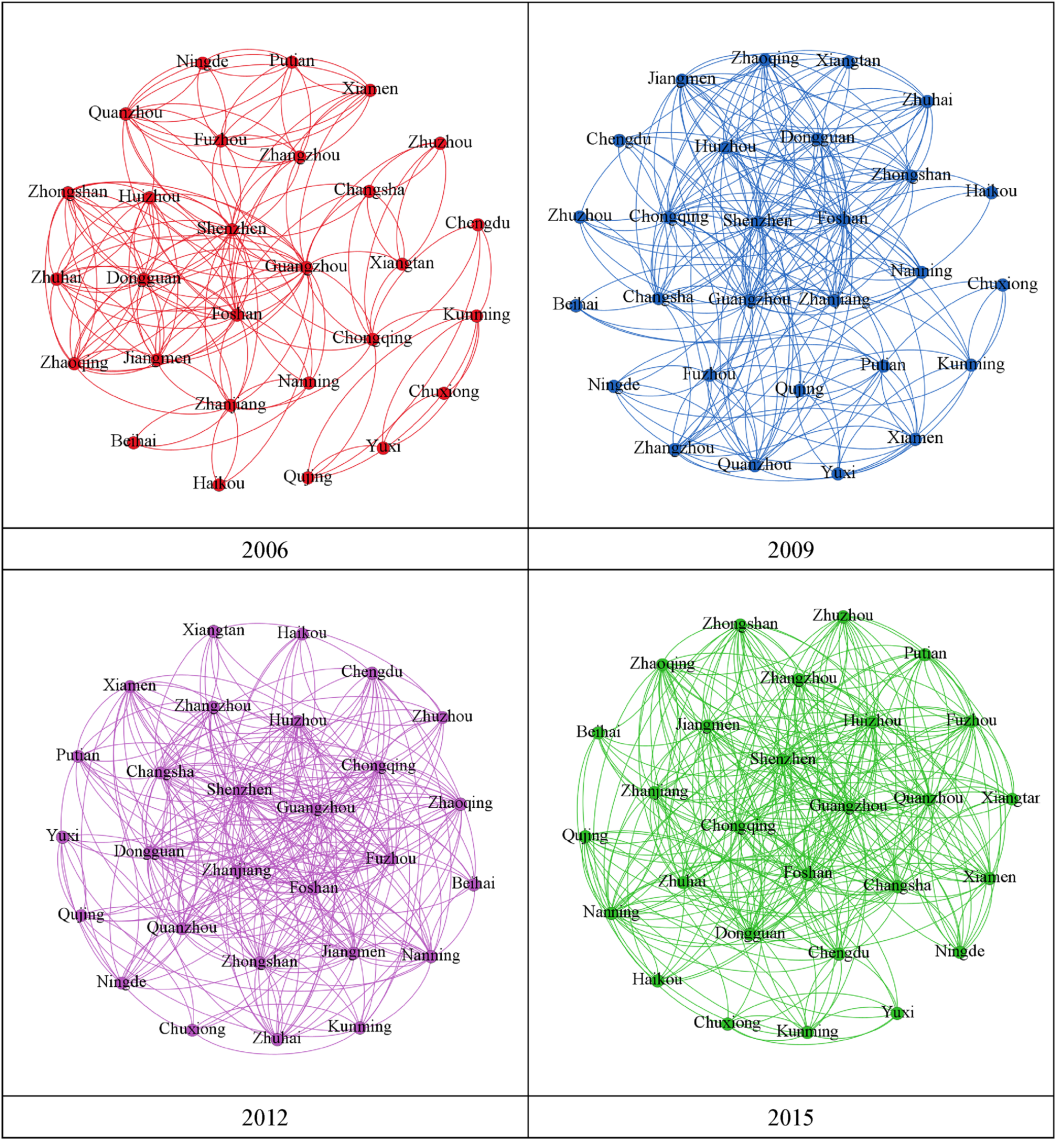
The evolution of the traffic correlation network structure in the Pearl River Delta (2006, 2009, 2012, 2015).

In the course of ten years of evolution, as shown in Fig. 2, the intercity communication within the Pearl River Delta Urban Agglomeration has gradually increased, and the traffic connection network of the urban agglomeration has formed a complex interactive cross-regional pattern in 2015 from the adjacent communication pattern at the core edge in 2006, with the resource elements showing the characteristics of agglomeration from west to southeast with the Pearl River Delta as the growth pole. Among them, the high flow intensity of internal factors in the Pearl River Delta Urban Agglomeration forms regional agglomeration, and there are three horizontal bands between the west coast of the Taiwan Strait and the central Yunnan urban agglomeration along the traffic connection network. The Sichuan-Chongqing Urban Agglomeration is on the network of elements, which mainly shows the characteristics of absorbing elements of surrounding urban agglomerations, and there are significant differences in urban development. The urban nodes of the Changsha-Zhuzhou-Xiangtan Urban Agglomeration play an intermediary role in the process of factor flow, which promotes the overall factor flow of the Pearl River Delta Urban Agglomeration and acts as a bridge for communication between the Pearl River Delta Urban Agglomeration and the Sichuan-Chongqing Urban Agglomeration.

**Figure 3:**
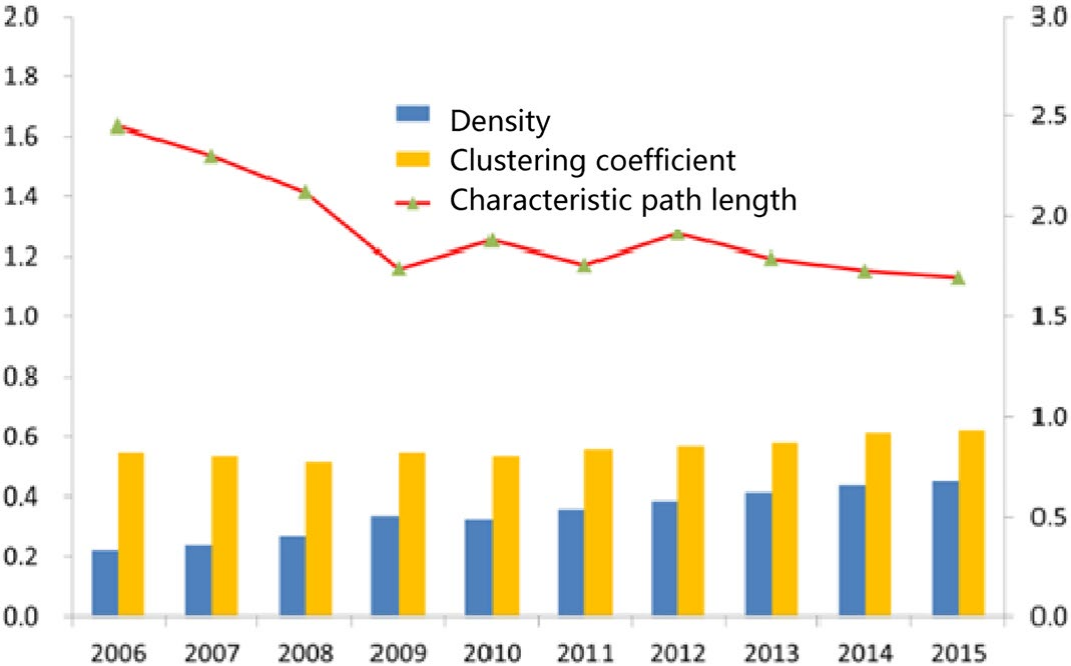
The overall characteristic index of the Pearl River Delta traffic correlation network (2006–2015). *Notes*: See^31^ for the path measurement method of the density, clustering coefficient and path length of the network.

From the change in network density (Fig. 3), the network density of Pearl River Delta Urban Agglomeration increases from 0.203 to 0.397 in 2015, and the increasing network density indicates that the degree of association among members is gradually increasing, which also means that the overall urban agglomeration transportation network has an increasing influence on individuals. That is, the construction of a transportation network can affect the flow path of elements between cities to promote exchanges and cooperation among node cities and realize economic growth. On the other hand, the clustering coefficient of the Pearl River Delta traffic correlation network gradually increases, and the characteristic path length gradually decreases, showing the basic characteristics of a small world network. The average characteristic path length decreased from 2.449 in 2006 to 1.695 in 2015, which indicates that the transaction cost of elements among cities decreased, the circulation speed of elements among cities became increasingly faster, and the efficiency of information and element flow within urban agglomerations continued to increase. However, it also indicates that there were a few key nodes and routes in the network that were under the pressure of main circulation, diversion and transit. Therefore, identifying key nodes and strengthening the links among cities became important to improve the efficiency of element flow in the Pan Pearl River Delta Urban Agglomeration. The promotion of the clustering coefficient indicates that the attraction between cities and the aggregation of elements promote the increase in traffic demand in node cities to promote the scale effect of traffic-related networks and better promote information transmission and element flow, so this effective benefit attracts cities to further enhance traffic construction in the network.

### Node attributes of the urban flow network

To reveal the position of different node cities in the transportation network of the Pearl River Delta Urban Agglomeration, this paper measures the degree centrality, betweenness centrality and closeness centrality of cities in the Pearl River Delta Urban Agglomeration in 2015. The research results are shown in Table 2 below. According to the node degree, describing the ability of resource aggregation and diffusion and the ability of resource transmission, this paper finds that the cities of the Pearl River Delta Urban Agglomeration can be summarized into the following four categories:

**Table 2:** The degree analysis of the traffic correlation network structure in the Pearl River Delta (2015).

City	Degree centrality		City	Betweenness centrality	City	Closeness centrality
	in-degree	out-degree				
Chongqing	27	7	**Shenzhen**	92.755	*Chongqing*	100.000
Guangzhou	25	17	**Kunming city**	55.276	*Guangzhou*	93.103
Shenzhen	25	20	**Guangzhou**	50.748	*Shenzhen*	93.103
Foshan	22	18	Chengdu	41.257	*Foshan*	84.375
Changsha	22	14	Chongqing	40.997	*Changsha*	84.375
Quanzhou	17	16	Foshan	37.593	Quanzhou	77.143
Fuzhou	16	12	Zhanjiang	28.495	Huizhou	75.000
Nanning	16	12	Changsha	27.832	Dongguan	72.973
Huizhou	16	17	Quanzhou	24.729	Jiangmen	72.973
Jiangmen	15	14	Nanning	21.948	Zhaoqing	72.973
Chengdu	15	8	Fuzhou	16.594	Fuzhou	72.973
Dongguan	14	17	Qujing city	13.688	Nanning	72.973
Xiamen	12	12	Huizhou	10.588	Zhongshan	71.053
Zhuzhou	12	9	Xiamen	9.878	Chengdu	69.231
Zhanjiang	12	14	Zhangzhou	7.671	Zhangzhou	69.231
Zhaoqing	11	16	Dongguan	7.207	Zhanjiang	69.231
Zhongshan	11	16	Jiangmen	6.472	Zhuhai	65.854
Zhangzhou	10	15	Zhuzhou	5.218	Zhuzhou	65.854
Zhuhai	9	13	Zhaoqing	4.437	Xiamen	65.854
Kunming city	7	8	Zhongshan	3.216	Putian	62.791
Putian	5	11	Putian	0.236	Xiangtan	60.000
Ningde	5	9	Zhuhai	0.165	Ningde	60.000
Qujing city	4	9	Xiangtan	0	Qujing city	60.000
Xiangtan	3	9	Ningde	0	Beihai city	60.000
Chuxiong city	3	5	Yuxi city	0	Kunming city	58.696
Yuxi city	3	5	Chuxiong city	0	seaport	58.696
Seaport	2	7	Seaport	0	Yuxi city	55.102
Beihai city	0	9	Beihai city	0	Chuxiong city	55.102

Bold indicate that the betweenness centrality of nodes is higher than 50, which means that these nodes are the hub nodes of the urban agglomeration. Unicom reflects the control ability of nodes in information transmission. Italic indicate that a node's closeness centrality is greater than 80, which indicates that the node has strong information transmission ability and reflects the independence of the node in the process of information transmission.

The first category is the core cities, represented by Guangzhou, Shenzhen and Foshan. These cities generally have a high degree of factor exit and entry. They not only have a remarkable ability to gather resource elements but also have obvious diffusion effects. They can effectively promote the economic growth of surrounding cities while meeting their own demand for high-quality development. Moreover, these cities have a high betweenness centrality and a strong ability to transfer resources, enabling them to play a positive role as a bridge, link the traffic conditions between cities and promote the efficient flow of information and elements, so they are at the core of the whole Pan Pearl River Delta Urban Agglomeration. The second category is the traffic gathering cities, represented by Chongqing and Chengdu. The obvious feature of these cities is that they have a high in-degree but a low out-degree. The reason is that these cities mainly rely on favorable government policies, absorb resources from surrounding cities, and achieve their own economic growth through the polarization effect. However, the imperfection of the regional transportation network has led to the fact that the city has not achieved diffusion and feedback. Although some cities have a high degree of centrality, more of them exert control over resources. The third category is the subcore cities, represented by Changsha, Quanzhou, Fuzhou, Nanning and Kunming. As the core of suburban agglomerations, these cities are in the middle and upper levels overall. However, these cities have a high centrality degree, such that they serve as the medium of resource flow between urban agglomerations and undertake the information element exchange between core cities and marginal cities. In the communication between core cities and marginal cities, they can realize the process of receiving and learning elements and information and maintain them. The fourth category is the marginal cities, represented by Beihai, Qujing, Chuxiong and Haikou. The factor entry and exit of these cities are low, and their low-level development restricts their ability to gather resources. Moreover, these cities are located in the periphery of the urban agglomeration, and the transportation infrastructure is extremely imperfect, which leads to the poor resource flow and low efficiency.

## Economic growth effect of the transportation network in the urban agglomeration

### Economic growth effect of network structure

As shown in Table 3, the regression coefficients of the network density, clustering coefficient and characteristic path length of the transportation-related network structure are 2.0560, 7.1290 and − 3.4607, respectively, which indicates that the network structure of the urban agglomeration transportation network has a significant impact on the coordinated development of the urban agglomeration. The increase in the network density and network clustering coefficient and the decrease in the characteristic path can effectively improve the degree of economic connection among cities within the urban agglomeration to promote the effective economic growth of the urban agglomeration. Therefore, we should continue to increase the intercity communication among cities in the urban agglomeration network, that is, increase the scale of the traffic connection network, which requires continuing to exert the degree of communication between governments, reducing policy barriers, and rationally adjusting the industrial distribution layout to continuously strengthen the spatial connection of the whole Pan Pearl River Delta Urban Agglomeration transportation network, effectively promote the flow of information and elements within the urban agglomeration transportation connection network, and promote the economic growth of the urban agglomeration.

**Table 3:** The overall effect of the traffic correlation network structure in the Pearl River Delta.

	(1)	(2)	(3)
Density	2.0560***	–	–
Clustering Coefficient	–	7.1290***	–
Distance	–	–	3.4607***
Constant	1.4524***	3.3287***	1.4598***
R-squared	0.5674	0.4003	0.4320

***, ** and * indicate that the significance levels are 1%, 5% and 10%, respectively.

### Economic growth effect of network nodes

From the regression results of the Pan Pearl River Delta Urban Agglomeration and sub-agglomerations (as shown in Table 4 below), before and after adding network factors, the coefficients of structural factors changed little, indicating that the model is robust. From the empirical analysis (models ii, iv, vi, viii, and x), the coefficients of these four variables are positive for the whole Pan Pearl River Delta Urban Agglomeration. Among them, the elasticity coefficients of in-degree and out-degree are 0.0147 and 0.0050, respectively, and the elasticity coefficients of closeness centrality and betweenness centrality are 0.024 and 0.0037, respectively. This shows that for the whole Pan Pearl River Delta Urban Agglomeration, the improvement in the individual transportation network structure promotes the economic growth of the urban agglomeration. This is because the transportation network is the key path of factor flow, and the traffic improvement of individual cities increases the likelihood of a city having connections with other cities and increases the frequency of the factor flow. On the one hand, it strengthens the opportunities for direct communication between the marginal cities and the core cities; on the other hand, it also expands the nodes that play an intermediary role, giving full play to their ability to gather and spread resources and promoting the economic development of surrounding cities while promoting their own economic development.

**Table 4:** The individual effects of network structure in the Pan Pearl River Delta Urban Agglomeration.

Independent variable	Pearl River		Pearl River Delta		West coast of Taiwan Strait		Beibu Gulf		Changsha, Zhuzhou and Xiangtan, central Yunnan	
	I	II	III	IV	V	VI	VII	VIII	IX	X
SIR	3.6972***	2.4863*** (3.90)	9.2968**	10.4370*** (5.41)	− 6.6798	− 7.1994** (− 2.54)	1.0404	0.4218 (0.58)	3.8543***	3.1406*** (3.15)
TIR	3.8749***	2.5148*** (4.12)	9.1805**	9.8595*** (5.38)	− 8.3359*	− 6.8315** (− 2.50)	2.8722***	1.0951* (1.66)	4.9852***	3.5667*** (3.40)
UR	1.3709***	0.8342*** (5.27)	0.1292	0.1683* (1.44)	4.8275***	1.0455 (1.29)	− 3.0145***	− 0.1437 (− 0.28)	2.2927***	2.7055*** (5.62)
FDI	0.2564***	0.0115*** (6.44)	0.0484***	0.0196*** (6.67)	0.0233	0.0347 (0.97)	0.1094***	0.0352*** (3.25)	0.0175***	0.0085*** (4.29)
Outdegree		0.0147* (1.61)		0.0252* (1.41)		0.0365** (2.07)		0.0506*** (4.14)		0.0281** (2.28)
Indegree		0.0050* (0.57)		− 0.0085 (− 0.57)		− 0.0187 (− 0.65)		0.0188 (0.71)		− 0.0530*** (− 3.05)
Closeness		0.0240*** (4.92)		0.0312*** (3.48)		0.0487*** (3.36)		0.0154 (1.40)		0.0079 (1.21)
Betweenness		0.0037*** (5.70)		0.0015** (2.47)		0.0022 (0.91)		0.0063** (3.41)		0.0055*** (4.36)
Constant	2.5764***	2.5852***	− 2.3724	− 5.1643***	12.4244***	10.3290***	6.1008***	4.7799***	1.4636***	2.0258***
Time trend	Fixed	Fixed	Fixed	Fixed	Fixed	Fixed	Fixed	Fixed	Fixed	Fixed
Individual effect	Fixed	Fixed	Fixed	Fixed	Fixed	Fixed	Fixed	Fixed	Fixed	Fixed
R-squared	0.7250	0.8736	0.8075	0.9424	0.3054	0.6861	0.8222	0.9309	0.8785	0.9357
Observations	280	280	90	90	40	40	60	60	90	90

***, ** and * indicate the significance levels of 1%, 5% and 10%, respectively.

Comparing the aggregation and diffusion effects of factor flows in different urban agglomerations under the structure of the traffic correlation network and finding effective solutions to achieve regionally coordinated growth under the premise of individual growth are the focus of this paper.

1. For the Pan Pearl River Delta Urban Agglomeration, the SIR, TIR and UR coefficients of model IV are higher than those of model III, which indicates that the industrial structure and urbanization rate based on factor flow can better promote the economic development of the Pan Pearl River Delta Urban Agglomeration, and the transportation network can reasonably improve the path of factor flow and realize the economic growth of the urban agglomeration. The transportation network structure, complementary to the rational division of labor of industrial structure in the Pearl River Delta Urban Agglomeration, drives urban economic growth. Moreover, the perfect transportation network of the Pearl River Delta Urban Agglomeration promotes the transfer efficiency of factors between cities, accelerates the movement of the population from agricultural to urban employment, and reduces the influence of foreign direct investment (FDI) on economic growth, which is the embodiment of China's major strategic decision to integrate economically into Eurasia.
2. For the Beibu Gulf City Group and Chang-Zhu-Tan-Chuan-Yu City Group, after adding the influencing factors of the transportation network (models i and ii, vii and viii, ixx and x), the transportation-related network has a positive impact, but the industrial structure, urbanization rate and the coefficient of foreign investment have decreased because the construction of the transportation network in these subcity groups is not enough to promote effective factor flow. On the other hand, there is irrational resource allocation in these urban agglomerations, and the industrial structure between cities tends to be homogeneous, which hinders the economic growth of the urban agglomerations. Moreover, under the influence of the transportation network, the urbanization rate accelerates the impact on the economic growth of urban agglomerations, and weakens the impact of FDI on economic growth. This is because the population and resource elements can achieve the goal of economic growth without more foreign investment under the agglomeration and diffusion effect between cities, relying on the path of the transportation network and taking the rational distribution of urban industries as the premise. Therefore, for these two urban agglomerations, we should continue to adjust the urban industrial structure on the basis of clarifying urban functions, promoting communication between noncentral cities and central cities, improving the transportation network, accelerating the pace of noncore (marginal) cities’ integration into the core circle, and building a multicenter and multifunctional urban system with core cities (Changsha, Fuzhou, Xiamen, Chongqing) as the mainstay and other cities as the auxiliaries.
3. Compared with other urban agglomerations, there is a highly negative correlation between the industrial structure of the secondary and tertiary industries on the west coast of the straits (v and v) and the economic development within urban agglomerations, which shows that the process of enhancing and upgrading the industrial structure of urban agglomerations cannot effectively promote economic growth. This is due to the blind pursuit of the upgrading of industrial structure along with the neglect of the rationalization of the industrial structure of urban agglomerations. In the model of urban flow intensity combined with the previous analysis, in the urban agglomeration on the west coast of the Taiwan Strait, the urban flow intensity of each city is much lower than that of other cities, which shows that the industries in the urban agglomeration are not closely connected, and the flow of resource elements among cities is not frequent. The development of Guangxi, Yunnan and Sichuan Provinces in the urban agglomeration on the west coast of the Taiwan Strait is relatively slow, and there are complex policy barriers between provinces and cities, which leads to the homogenization of industries among cities in the formed industrial clusters and makes cities more competitive than cooperative. Therefore, compared with other urban agglomerations, the urban agglomerations on the west side of the Taiwan Strait need to consider how to rationalize the upgrading of industrial structure. They should give full play to the intermediary role of central node cities, such as Nanning, Kunming and Chengdu, establish a multicore urban agglomeration network, adjust the rational division of labor among different cities by relying on their abundant natural resources, establish a complementary mechanism of urban functions, and improve the efficiency of the spatial aggregation and diffusion of elements to deepen the economic ties between urban agglomerations on the west side of the Taiwan Strait and other urban agglomerations and ensure healthy economic development in urban agglomerations.

## Conclusions and implications

This paper analyzes the spatial evolution pattern and network characteristics of the transportation network of the Pan Pearl River Delta Urban Agglomeration and studies the influence of the transportation network on the economic growth of the urban agglomeration. The main conclusion is as follows:

1. The overall structure of the transportation network of the Pan Pearl River Delta Urban Agglomeration has obvious characteristics of a small world network, showing a typical center-edge structure and gradually evolving to a multicenter transportation network. The density and aggregation coefficient of the network are increasing year by year, and the number of isolated cities in the network is decreasing, which means that the communication between cities in the Pan Pearl River Delta Urban Agglomeration is becoming closer. The reduction in the shortest path of information transmission and factor flow between cities and the reduction in transaction costs can promote the frequent flow and transmission efficiency of resource factors in urban networks. From the analysis of the characteristics of a single network structure, the aggregation and diffusion of resource elements in the transportation network of the Pan Pearl River Delta Urban Agglomeration lead to significant differences in the development of urban nodes.
2. The increase in network density and the network aggregation coefficient and the decrease in the characteristic path of the traffic-related network in the Pan Pearl River Delta Urban Agglomeration can effectively promote the economic ties among cities in a city group. This shows that the improvement in the individual traffic network structure enables more node cities to effectively support the aggregation and diffusion of elements and act as bridges of element transmission, resulting in more frequent exchange of elements among cities and promoting the economic growth of urban agglomerations. In addition, the improvement in transportation network connectivity has stimulated the vitality of regional industry and investment, indirectly promoting economic growth. However, the impact of the transportation network on economic growth also shows regional heterogeneity, which depends on whether the internal industrial structure layout of suburban agglomerations can reasonably absorb resource elements to achieve cooperation rather than competition and whether the degree of transportation links between cities can maximize the efficiency of factor flow.

The above conclusions yield the following policy implications:

1. From the perspective of urban agglomerations as a whole, mega-urban agglomerations contain a large number of urban individuals with different resource endowments and development patterns. Transportation is a physical bridge to promote information exchange and industrial cooperation among these cities and finally form integrated development. Therefore, it is a priority to further strengthen the construction of transportation infrastructure in mega-city groups. Of course, this kind of investment is not extensive, so we should give full consideration to the position of cities in urban agglomerations and make appropriate investments. For example, marginal cities need to increase investment in urban transportation infrastructure, rationalize the industrial layout, make effective use of the intermediary role of surrounding semicore cities, and strengthen ties with core cities. Moreover, cooperation between local governments is a necessary supporting measure to lower the administrative barriers between cities and avoid the hidden obstacles of local protectionism to the factor flow.
2. From the perspective of internal suburban agglomerations in the Pan Pearl River Delta Urban Agglomeration, the intercity transportation network has become complete, and the industrial layout has gradually reached a high quality. The factor aggregation effect is obvious enough, and increased aggregation may support intercity diffusion, the export of surplus factors and practical experience and economic development in the surrounding areas. For the Beibu Gulf and Changsha-Zhuzhou-Xiangtan-Sichuan-Chongqing Urban Agglomerations, the internal industrial layout is more reasonable. The government should pay more attention to strengthening the communication between noncentral cities and central cities, improving the transportation network, and speeding up the process of integrating noncore (marginal) cities into the core circles. For the urban agglomerations on the west side of the straits, we should give full play to the intermediary role of Nanning, Kunming and Chengdu, establish a multicore urban agglomeration network, and improve the efficiency of the spatial aggregation and diffusion of elements to deepen the economic ties between the urban agglomerations on the west side of the straits and other urban agglomerations and realize the coordinated development of the internal economy of the Pearl River Delta Urban Agglomeration.

Finally, our research mainly analyzes the influence of traffic networks on economic growth across the whole urban agglomeration, but we have not paid attention to the heterogeneous structure of the network and the specific differences in internal development modes in different regions. In addition, we have not revealed the internal mechanism by which the traffic network affects economic growth. This can be achieved with more detailed research and case analysis in the future.

## Data Availability

The datasets of cities economics and society generated and analysed during the current study are available in the http://www.pprd.org.cn/?mobile and http://www.stats.gov.cn/ repository. The data of environment and emission that support the findings of this study are available from EPS dataset but restrictions apply to the availability of these data, which were used under license for the current study, and so are not publicly available. Data are however available from the authors upon reasonable request and with permission of EPS (https://www.epsnet.com.cn/index.html#/Index).
